# Supplementary material for: Chopping the tail: how preventing superspreading can help to maintain COVID-19 control
Source: medRxiv. 2020 Jul 3:2020.06.30.20143115. Preprint. [Version 1] doi: 10.1101/2020.06.30.20143115 (PMC7340192; doi:10.1101/2020.06.30.20143115)
Supplement: 1 [file NIHPP2020.06.30.20143115-supplement-1.pdf]

medRxiv preprint doi: https://doi.org/10.1101/2020.06.30.20143115.this version posted July 3, 2020. The copyright holder for this preprint (which was not certified by peer review) is the author/funder, who has granted medRxiv a license to display the preprint in perpetuity.
It is made available under a CC-BY 4.0 International license .

Supplemental Material for "Chopping the tail: how preventing superspreading can help to maintain COVID-19 control"

Morgan P. Kain[1,2]*, Marissa L. Childs[3]*, Alexander D. Becker[1], Erin A. Mordecai[1]

*Denotes equal authorship. Corresponding authors: morganpkain@gmail.com, marissac@stanford.edu

[1]Department of Biology, Stanford University, Stanford, CA, 94305, USA

[2]Natural Capital Project, Woods Institute for the Environment, Stanford University, Stanford, CA 94305, USA

[3]Emmett Interdisciplinary Program in Environment and Resources, Stanford University, Stanford, CA, 94305, USA

medRxiv preprint doi: https://doi.org/10.1101/2020.06.30.20143115.this version posted July 3, 2020. The copyright holder for this preprint (which was not certified by peer review) is the author/funder, who has granted medRxiv a license to display the preprint in perpetuity.
It is made available under a CC-BY 4.0 International license .

Figures

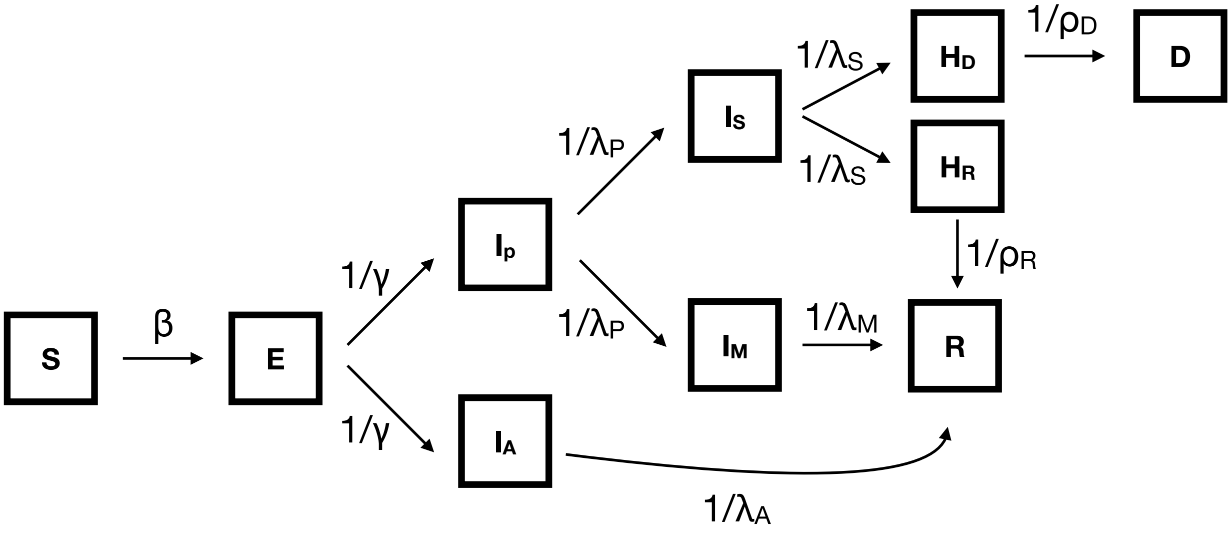

State variables

S = Susceptible
E = Exposed
I_P = Pre-symptomatic
I_A = Asymptomatic
I_S = Symptomatic, severe case
I_M = Symptomatic, mild case
H_D = Hospitalized, eventual death
H_R = Hospitalized, eventual recovery
R = Recovered
D = Dead

Transition Rates

β = Transmission rate
γ = Preinfectious period
λ_A = Asymptomatic infectious period
λ_P = Presymptomatic infectious period
λ_S = Severe infectious period until hospitalization
λ_M = Mild infectious period
ρ_D = Hospitalization period until death
ρ_R = Hospitalizations period until recovery

Figure S1: Epidemiological model box diagram

medRxiv preprint doi: https://doi.org/10.1101/2020.06.30.20143115.this version posted July 3, 2020. The copyright holder for this preprint (which was not certified by peer review) is the author/funder, who has granted medRxiv a license to display the preprint in perpetuity.
It is made available under a CC-BY 4.0 International license .

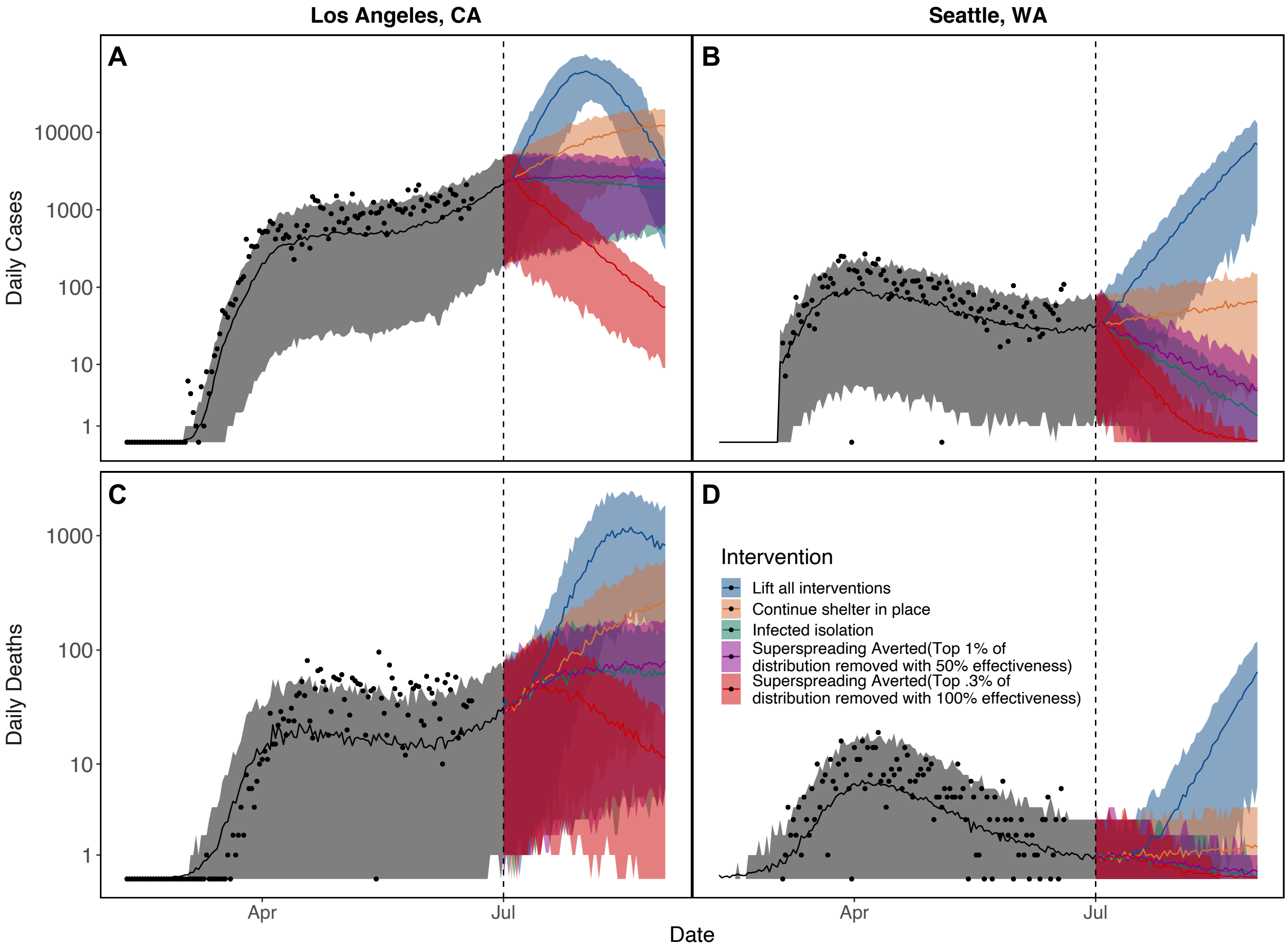

Figure S2: Many other truncation interventions are viable alternatives to the top 0.5% with 75% efficiency presented in the main text including: truncating the top 1% with 50% efficiency (purple) and top 0.3% with 100% efficiency (red). Bands show 95% CI on stochastic simulations of daily cases and deaths for the single maximum likelihood estimate. Dates range from February 2020 to October 2020.

medRxiv preprint doi: https://doi.org/10.1101/2020.06.30.20143115.this version posted July 3, 2020. The copyright holder for this preprint (which was not certified by peer review) is the author/funder, who has granted medRxiv a license to display the preprint in perpetuity.
It is made available under a CC-BY 4.0 International license .

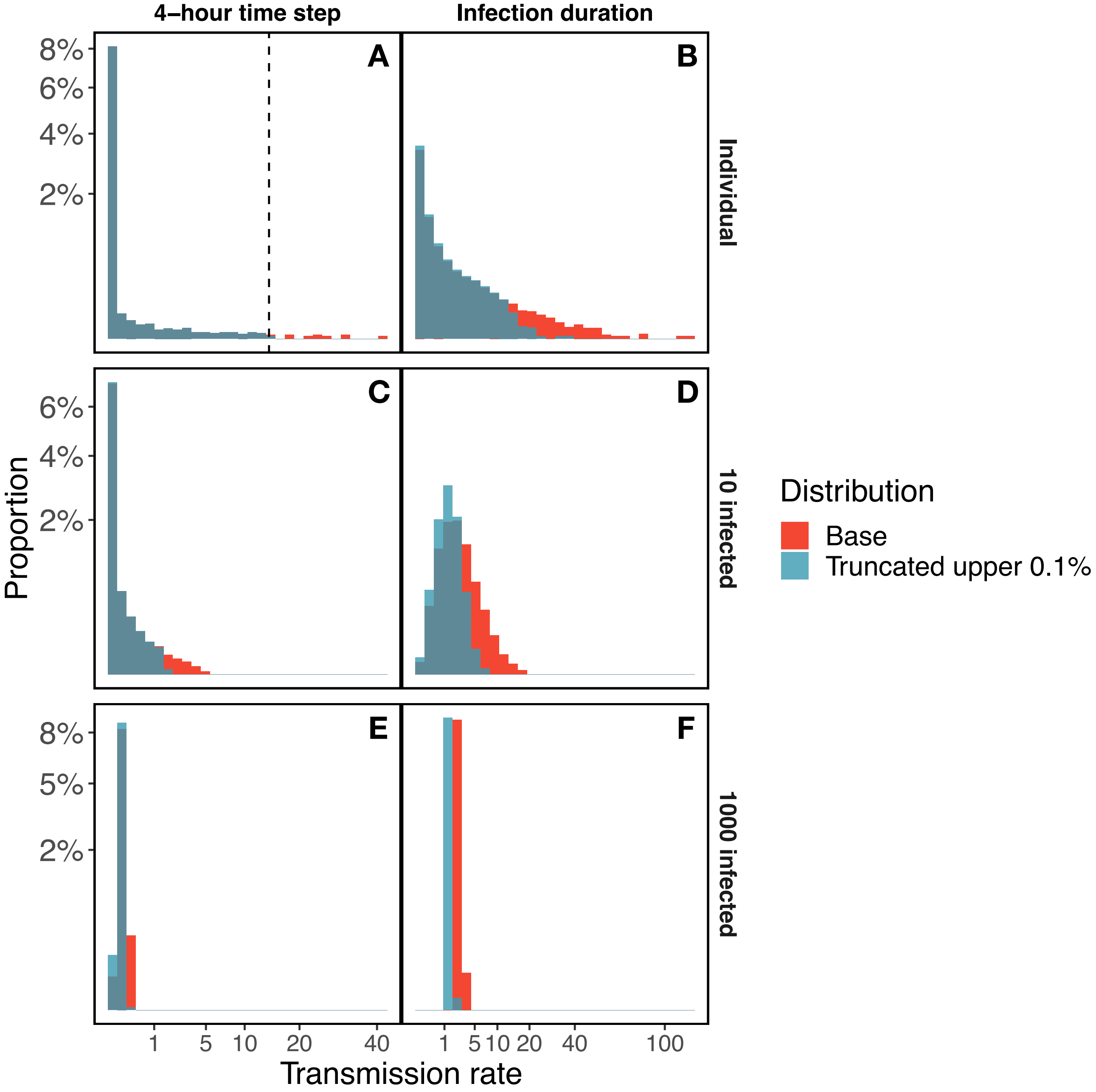

Figure S3: Expanded view of truncating the upper 0.1% of the individual level time step transmission rate distribution (π) at a four-hour time step (A). This truncation leads to a reduction of the mean and variance for an individual's infectious period reproduction potential (B). As the number of infected individuals in the population increases from 10 (C, D) to 1000 (E, F), the variance in decreases in both the population-level average transmission rate during each 4-hour period (C, E) and over the lifetime of those infected (D, F).

medRxiv preprint doi: https://doi.org/10.1101/2020.06.30.20143115.this version posted July 3, 2020. The copyright holder for this preprint (which was not certified by peer review) is the author/funder, who has granted medRxiv a license to display the preprint in perpetuity.
It is made available under a CC-BY 4.0 International license .

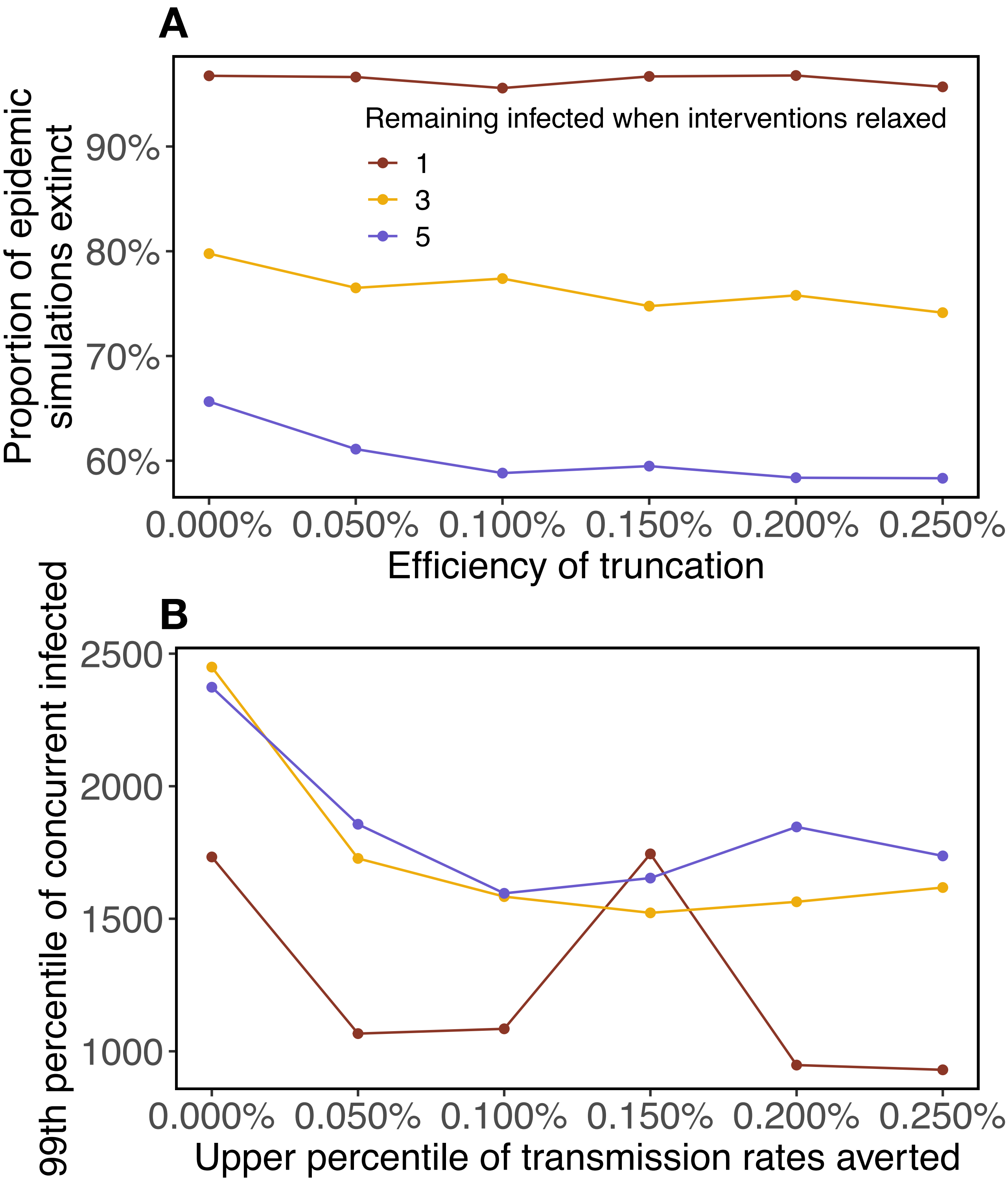

Figure S4: The proportion of epidemic simulations that went extinct (A) and the upper 99th percentile of the number concurrent infected after 42 days (B) for the resurgent simulations among 5000 total simulations for increasing truncation proportions of π. Shelter-in-place is scaled so that transmission rate at the time of intervention relaxation is identical across intervention scenarios and would result in $\mathcal{R}_E = 2$ in a fully susceptible population.

medRxiv preprint doi: https://doi.org/10.1101/2020.06.30.20143115.this version posted July 3, 2020. The copyright holder for this preprint (which was not certified by peer review) is the author/funder, who has granted medRxiv a license to display the preprint in perpetuity.
It is made available under a CC-BY 4.0 International license .

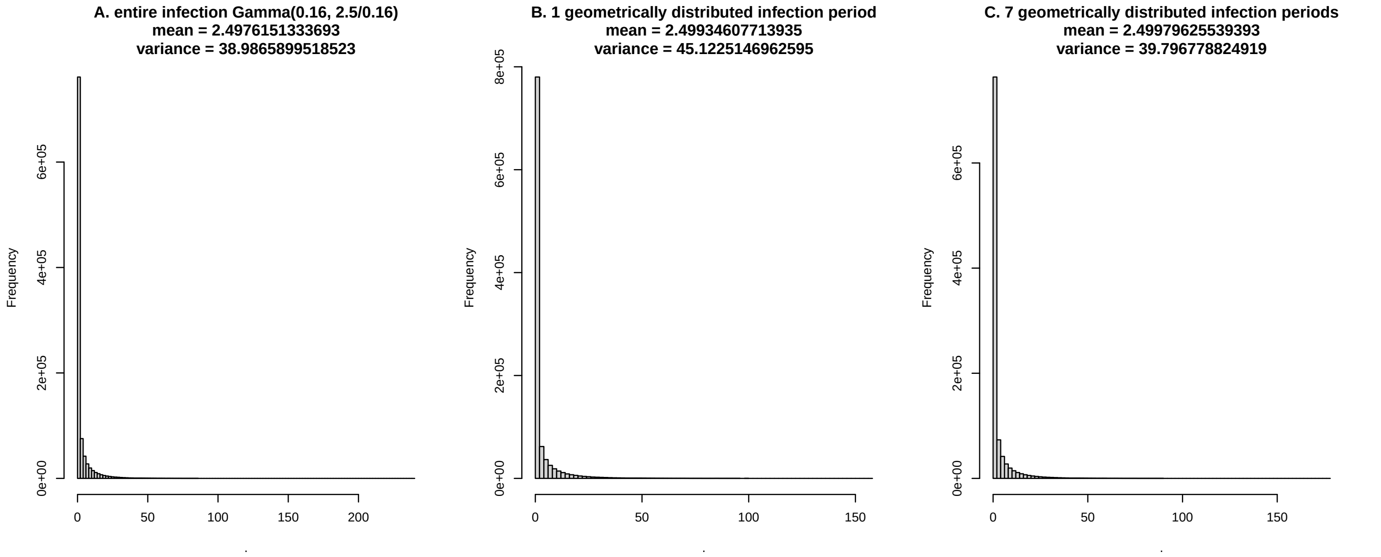

Figure S5: The distribution of individual lifetime reproduction (R) when modeled as a Gamma distribution with a mean of 2.5 and a scale of 0.16 (A). This distribution implicitly assumes a constant infectious duration. Using a geometrically distributed infectious period with only one period ("box"), and a time period of 4 hours result in an increase in the variance of the individual reproductive distribution relative to assuming a constant infectious period (B). Breaking the infectious period into 7 sub-stages (boxes) reduces the variance, though the variance remains marginally higher than when assuming a constant infectious period (C).
